# Utility of the angle between the cervical canal and the anatomical conjugate line for predicting pouch of Douglas obliteration in patients with posterior placenta previa

**DOI:** 10.1371/journal.pone.0290244

**Published:** 2023-08-17

**Authors:** Satoshi Shinohara, Mayuko Kasai, Genki Yasuda, Rei Sunami

**Affiliations:** Department of Obstetrics and Gynecology, Yamanashi Prefectural Central Hospital, Kofu, Yamanashi, Japan; University of Bremen: Universitat Bremen, GERMANY

## Abstract

**Aim:**

Pouch of Douglas obliteration, which prevents exteriorization of the uterus, increases surgical morbidity in patients with placenta previa. We aimed to identify magnetic resonance imaging features that can predict pouch of Douglas obliteration preoperatively.

**Methods:**

We retrospectively assessed 39 women with posterior placenta previa who underwent magnetic resonance imaging for the preoperative assessment of placenta accreta spectrum. We defined the angle formed by the anatomical conjugate line (based on pelvimetry) and the cervical canal as the cervical inclination angle, which was measured on sagittal T2-weighted magnetic resonance imaging. Subsequently, we analyzed the correlation between the cervical inclination angle and pouch of Douglas obliteration.

**Results:**

The median maternal age was 34 years (range, 22–44 years) and 26 (66.7%) women delivered at term. The median cervical inclination angle was 98° (range, 71–128). Pouch of Douglas obliteration was confirmed in six patients (15.4%). The cut-off value of the cervical inclination angle for the prediction of pouch of Douglas obliteration was 102° with a sensitivity of 66.7%, specificity of 78.8%, positive predictive value of 36.4%, and negative predictive value of 92.9% (area under the curve, 0.83).

**Conclusions:**

Measuring the cervical inclination angle may help in ruling out an obliteration of the pouch of Douglas. It may also be useful in the operative management of women with posterior placenta previa. However, caution should be exercised when generalizing the results of this study because of the small sample size, which makes the results prone to bias.

## Introduction

Placenta previa is associated with significant intraoperative and postpartum bleeding [1, 2]. According to previous studies [3–5], the risk of placenta previa is higher in women with endometriosis than in those without endometriosis (6.0% versus 1.0%). Moreover, endometriosis is widely known as a major cause of posterior extrauterine wall adhesions [5]. Reliable predictors of massive hemorrhage and appropriate treatment strategies are vital for the successful management of placenta previa. Previous studies have reported that placenta accreta spectrum (PAS), advanced maternal age, history of previous cesarean section, and pouch of Douglas obliteration can serve as predictive factors of massive hemorrhage in women with placenta previa [6–8]. Various surgical methods, such as intrauterine balloon tamponade, uterine compression suture, and hysterectomy, have been used to control bleeding in women with abnormal placental location or placental invasion anomalies [9–12]. These surgical procedures usually entail exteriorization of the uterus, thereby raising the risk of intraoperative complications such as intestinal injury in women with pouch of Douglas obliteration. Magnetic resonance imaging (MRI) is usually used to assess the risk of pouch of Douglas obliteration before gynecological surgery. Several MRI findings associated with posterior extrauterine wall adhesions have been reported to date, including uterine retroflexion, displacement of intraperitoneal fluid, adhesion of bowel loops, and others [13]. However, these MRI findings were obtained in non-pregnant women, rendering them unsuitable for the assessment of the enlarged uterus during pregnancy. Moreover, risk assessment of pouch of Douglas obliteration in pregnant women has received little attention. According to a previous study [5], posterior extrauterine adhesions are more likely to be present in cases of placenta previa where the cervical canal is visualized horizontally. MRI scans of all patients included in their study were composed of 1.5-T superconducting magnets manufactured by General Electric (GE Healthcare, Waukesha, WI, USA), Philips (Philips North America, Andover, MA, USA), or Siemens (Siemens Corporation, Washington, DC, USA) [5]. Moreover, that study [5] reported that the cervical canal angle, which is defined as the angle between the line perpendicular to the broad of the back and the line passing through the internal os to the external os, was useful for predicting the absence of pouch of Douglas obliteration in women with placenta previa. Although this avenue is promising, it is possible that this method of measurement of the cervical canal angle is affected by maternal posture and soft tissues such as subcutaneous fat; therefore, it might not have high reproducibility. The line perpendicular to the broad of the back is important in calculating the cervical canal angle; however, correctly drawing the line of the broad of the back is relatively difficult due to maternal posture, soft tissues, and the extent of the back depicted on MRI. In other words, the cervical canal angle is at risk of being easily underestimated or overestimated, depending on the extent of the back depicted on MRI and the mother’s physique.” To overcome this issue, we devised a new measurement method that focuses on the inclination of the cervical canal relative to the anatomical conjugate line based on pelvimetry to predict pouch of Douglas obliteration. Assessment of pouch of Douglas obliteration is necessary for all pregnant women who are scheduled for a cesarean section. It is especially reasonable in women with placenta previa as they are likely to require surgical procedures to control bleeding, which may entail exteriorization of the uterus [2, 8–11]. Therefore, we conducted this retrospective study to evaluate the utility of the angle formed by the anatomical conjugate line (based on pelvimetry) and the cervical canal on MRI for predicting pouch of Douglas obliteration in patients with posterior placenta previa.

## Methods

### Study design

This retrospective observational cohort study was conducted at Yamanashi Prefectural Central Hospital between April 2016 and December 2021. We enrolled women with posterior placenta previa, including those with low-lying placenta as defined by a previous study [5], who underwent MRI for the preoperative assessment of PAS, delivered via cesarean delivery, and whose surgical records were available. Women with multiple pregnancies and premature rupture of fetal membranes (PROM) before MRI were excluded. Moreover, we excluded cases where gauze tamponade was performed as a hemostatic procedure for massive genital bleeding during MRI. The study protocol was reviewed and approved by the Human Subjects Review Committee of Yamanashi Prefectural Central Hospital (approval number: rinshou 2021–21), which waived the requirement for informed consent because of the retrospective design of this study. Nevertheless, the participants were provided with the opportunity to opt out of providing their data on the hospital’s website. All procedures were performed in accordance with the principles of the Declaration of Helsinki of 1964 and its subsequent amendments.

### Data collection

Obstetric data were collected from the medical and operative records. Gestational age was determined on the basis of the maternally reported last menstrual period and was confirmed by the crown-rump length measured on ultrasonography performed during the first-trimester. We recorded data on the mother’s age at delivery, orientation of the uterus in the early first trimester, gestational age at MRI examination, cervical length, history of previous cesarean section, use of in vitro fertilization, parity, amount of bleeding including amniotic fluid, gestational age at delivery, maternal stature, pre-pregnancy weight status, fetal sex, neonatal birth weight, presence of gestational diabetes mellitus (GDM), and hypertensive disorders of pregnancy (HDP). Placenta previa was defined as the presence of placental tissue extending over the internal cervical os [14]. Low-lying placenta was defined as the location of the inferior placental edge within 2 cm of the internal os but not overlying it [15]. HDP was defined as blood pressure ≥140/90 mmHg on at least two occasions during pregnancy [16]. The pre-pregnancy body mass index was calculated according to the World Health Organization standard [body weight (kg)/height (m)^2^]. The presence of ≥1 abnormal plasma glucose value (≥92, 180, and 153 mg/dL for fasting, 1-h, and 2-h plasma glucose concentrations, respectively) after a 75-g oral glucose tolerance test was diagnosed as GDM [17]. Cervical length was measured using transvaginal ultrasonography within one week of MRI examination. The orientation of the uterus was classified as retroflexed (S1 Fig) or non-retroflexed (anteverted or retroverted) (S2 and S3 Figs) based on transvaginal ultrasonography conducted during early pregnancy (from 5 to 6 weeks gestation), since a retroflexed uterus may be associated with posterior deep infiltrating endometriosis [18, 19].

**Fig 1:**
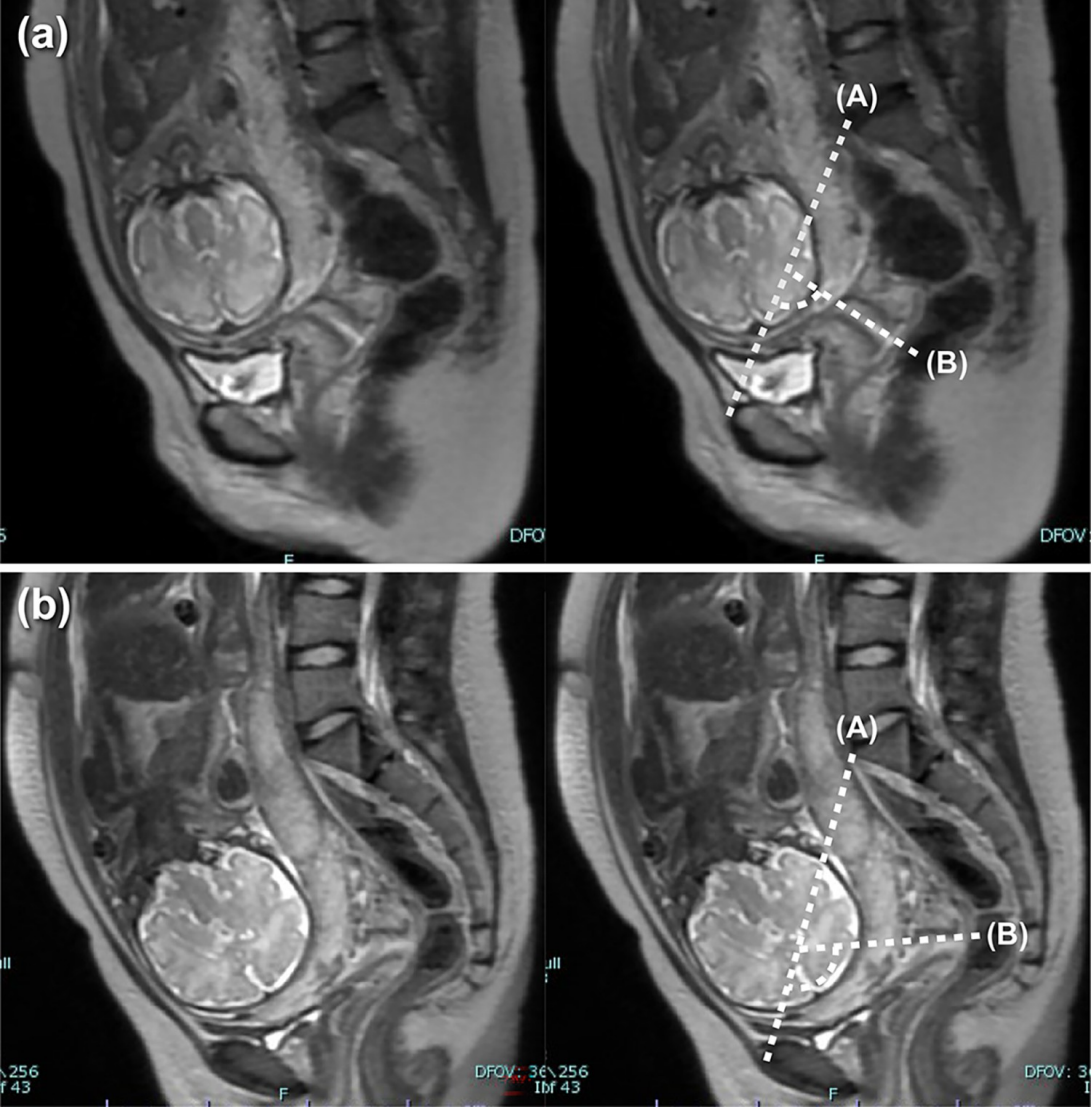
Magnetic resonance imaging (MRI) showing measurement of the cervical inclination angle. (a) Typical MRI findings in a patient without pouch of Douglas obliteration. (b) Typical MRI findings in a patient with pouch of Douglas obliteration.

We defined the angle formed by the anatomical conjugate line (based on pelvimetry) and the cervical canal as the cervical inclination angle (CIA), which was measured using MRI. MRI was performed at approximately 34 weeks gestation, since the mean reported gestational age at delivery in patients with placenta previa is 34–35 weeks [17]. However, the timing of MRI was left to the judgment of the treating obstetrician, depending on the presence of warning bleeding and signs of impending preterm labor. We projected a straight line through the anatomical conjugate line on the sagittal T2-weighted MRI scan (Fig 1A and 1B, Line A). Thereafter, we identified the line passing through the internal os to the external os (Fig 1A and 1B, Line B). We used a protractor to measure the angle between Lines A and B (Fig 1A and 1B), which was designated as the CIA (Fig 1A and 1B). The cervical canal angle was also calculated from the MRI scan, which has been described in detail in a previous study [5]. In all cases, two obstetricians (SS and RS), unaware of the women’s clinical characteristics and surgical findings, independently measured the CIA and cervical canal angle. The mean values were used for analysis.

Based on a previous study [5], we considered pouch of Douglas obliteration to be present if any of the following criteria were documented in the surgical records: (1) adhesions between the posterior extrauterine wall and the small bowel, colon, rectum, or pelvic wall; (2) exteriorization of the uterus was impossible because of posterior adhesions; and (3) adhesions were dissected for extracorporeal elevation.

All patients included in our study underwent MRI at Yamanashi Prefectural Central Hospital using two 1.5-T MR scanners (Signa HDxt; GE Medical Systems, DC, USA and Siemens Magnetom Symphony; Siemens AG, Erlangen, Germany).

### Statistical analysis

First, the Mann-Whitney U test and χ^2^ test were performed to compare the maternal and neonatal outcomes. Fisher’s exact test was used when the expected frequency was <5.

As the histograms were not normally distributed for several continuous variables, we selected the Mann-Whitney U Test, which can be used to test whether there is a difference between two groups, and the data need not be normally distributed. Second, receiver operating characteristic (ROC) curve analysis was conducted to determine the best cut-off value for the CIA and cervical canal angle. We used the Youden index, which describes the maximum vertical distance between the ROC curve and the diagonal (random chance), to estimate the optimal cut-off values [20]. Finally, we calculated the intraclass correlation coefficient (ICC) to evaluate the inter-rater reliability of the CIA and cervical canal angle. All analyses were performed using Bell Curve for Excel (Social Survey Research Information Co., Ltd., Tokyo, Japan) and IBM SPSS Statistics version 25 (IBM Inc., Tokyo, Japan). *p*-values <0.05 were considered statistically significant.

## Results

During the study period, 44 women with posterior placenta previa underwent MRI for the preoperative assessment of PAS. Thirty-nine women were included in this study, after excluding 5 women (twin pregnancy, *n* = 2; PROM, *n* = 1; strong cervical canal curvature, *n* = 1; gauze tamponade, *n* = 1). The median maternal age was 34 (range, 22–44) years; 15 (38.5%) women were nulliparous, and 26 (66.7%) had term deliveries. The median CIA was 98° (range, 71°–128°). The overall incidence pouch of Douglas obliteration was six (15.4%). In evaluating the orientation of the uterus, 32 of the 39 women with posterior placenta previa were included in this study, after excluding 7 women (data missing, *n* = 5; inappropriate for diagnosis, *n* = 2). Three (9.4%) of the 32 women had a retroflexed uterus. Although the sample size of the retroflexed group was too small for us to draw a conclusion, the frequency of pouch of Douglas obliteration (66.7% versus 6.9%, respectively; *p* = 0.04) differed significantly between the retroflexed (*n* = 3) and non-retroflexed groups (*n* = 29).

**Fig 2:**
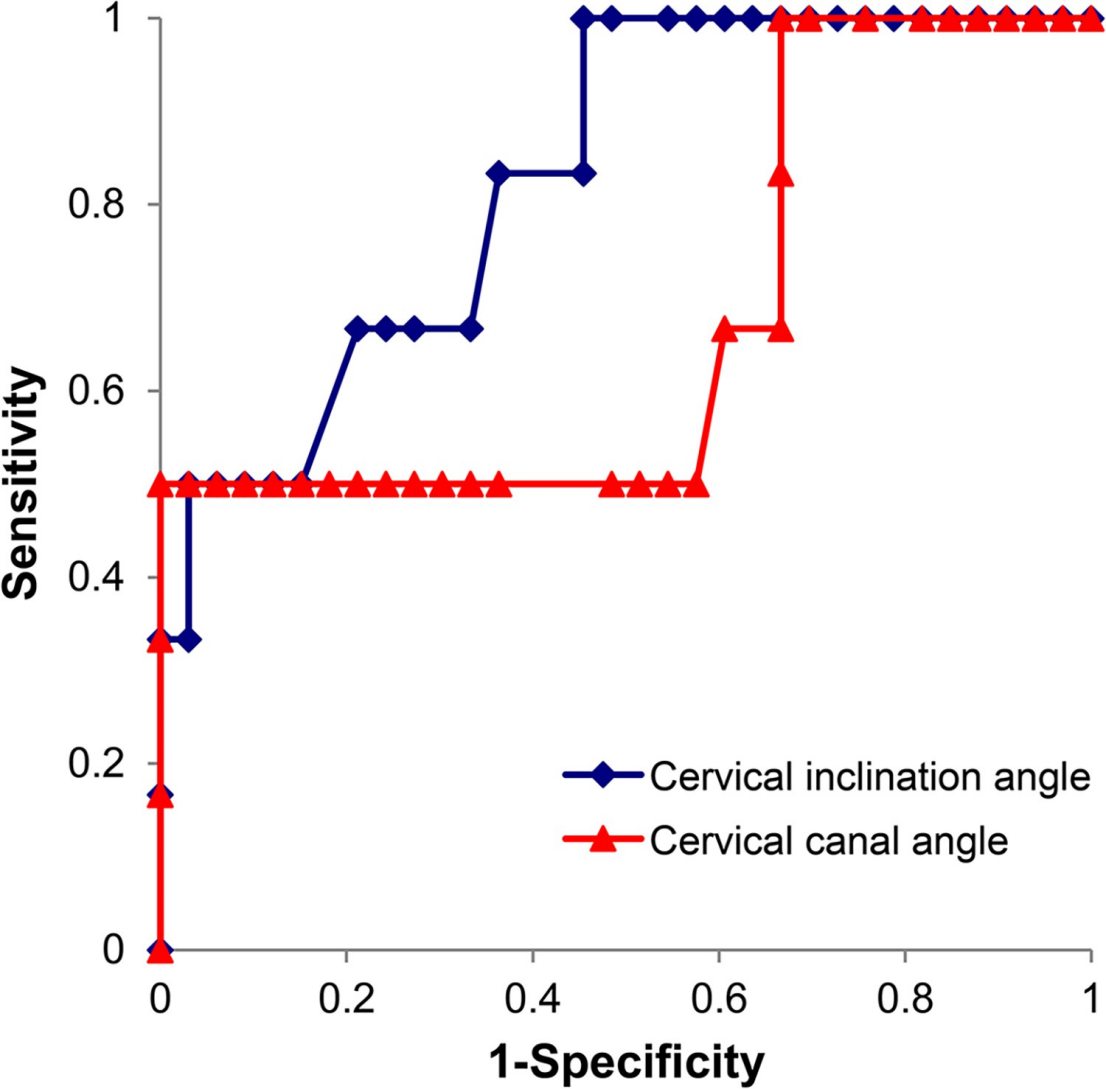
Receiver–operating curve analysis to determine the best cut-off value of the cervical inclination angle and cervical canal angle for predicting pouch of Douglas obliteration.

ROC analysis (Fig 2) revealed that the cut-off value of 102° for the CIA would allow for the maximum number of women to be correctly classified according to the extrauterine posterior adhesions. The sensitivity, specificity, positive predictive value (PPV), negative predictive value (NPV), and area under the curve (AUC) for this cut-off value were 66.7%, 78.8%, 36.4%, 92.9%, and 0.83, respectively.

The characteristics of CIA ≥102° and CIA <102° groups were similar, except that the frequency of pouch of Douglas obliteration was higher in the CIA ≥102° group and the cervical length was shorter in the CIA <102° group (Table 1). The inter-rater reliability for the CIA was relatively high with an ICC (2,1) of 0.80 (95% CI: 0.57–0.91). The inter-rater reliability for the cervical canal angle showed an ICC (2,1) of 0.64 (95% CI: 039–0.79).

**Table 1:** Baseline characteristics of the study population.

Characteristic	Cervical inclination angle <102° *n* = 28	Cervical inclination angle ≥102° *n* = 11	*p*-value
MRI examination, weeks	34 (27–35)	33 (31–35)	0.63
Maternal age, years	35 (22–44)	30 (27–40)	0.19
Cervical length, mm	33 (9–41)	36 (29–48)	0.03
Amount of bleeding at delivery, g	2011 (760–4600)	1930 (781–3020)	0.84
Birth weight, g	2629 (1798–3216)	2626 (2008–3166)	0.77
Gestational age at delivery	37 (31–38)	37 (35–38)	0.77
Male infant	16 (57.1)	5 (45.4)	0.51
History of previous cesarean section	3 (10.7)	1 (9.1)	0.88
ART	7 (25.0)	1 (9.1)	0.27
Nulliparity	11 (39.3)	4 (36.4)	0.87
Pre-pregnancy BMI, kg/m^2^	20.3 (17.3–25.4)	20.3 (17.6–24.2)	0.61
GDM	3 (10.7)	0 (0.0)	0.26
HDP	1 (3.6)	1 (0.9)	0.48
Pouch of Douglas obliteration	2 (7.1)	4 (36.3)	0.02
Retroflexed uterus^†^	2/23 (8.7)	1/9 (11.1)	1.00
Low-lying placenta	11 (39.3)	3 (27.2)	0.71

Values are presented as median (range) or number (%).

Abbreviations

MRI, magnetic resonance imaging

ART, assisted reproductive technology

BMI, body mass index

GDM, gestational diabetes mellitus

HDP, hypertensive disorders of pregnancy.

^†^The orientation of the uterus was examined in 32 patients. For more details, see the Results section.

## Discussion

In this study, we demonstrated that the CIA could be a useful predictor for pouch of Douglas obliteration in women with posterior placenta previa. Five (83.3%) of 6 women with posterior extrauterine adhesions in this study cohort did not have pelvic inflammatory disease or a history of pelvic surgery, which are known risk factors for posterior extrauterine adhesions [5], and were strongly suspected to have endometriosis based on intraoperative findings. A general consensus exists that endometriosis is among the major causes of abdominal adhesion [3–5].

Previous studies reported that the atypical orientation of the uterus, such as retroflexion, which reflects the adhesion between the posterior wall of the uterus and the retroperitoneum or intestinal tract due to endometriosis, pelvic inflammatory disease, and pelvic surgery, is associated with pouch of Douglas obliteration [18–22].

We examined the association between the atypical orientation of the uterus and obliteration of the pouch of Douglas using ultrasonography imaging performed in the early first trimester. We found that the frequency of pouch of Douglas obliteration was significantly higher in the retroflexed group than in the non-retroflexed group. However, as shown in Table 1, CIA values in late pregnancy were not always high in patients with a retroverted uterus in early pregnancy; thus, we infer that it is important to evaluate the CIA on MRI during late pregnancy to predict pouch of Douglas obliteration, irrespective of the orientation of the uterus during early pregnancy.

A previous study that conducted propensity score matching for patients with placenta previa reported that the amount of intraoperative blood loss was significantly higher in the posterior extrauterine adhesion group than in the control group [8]. Moreover, a recent systematic review showed that adhesions in the pelvis due to endometriosis were associated with a higher incidence of postpartum hemorrhage, hysterectomy, and bladder injury [23]. Therefore, predicting pouch of Douglas obliteration by measuring the CIA for patients with placenta previa is extremely crucial in the third trimester before cesarean section, in order to predict the level of difficulty of the surgery and prepare for appropriate treatment.

Matsuzaki et al. reported that the cervical canal angle is useful for predicting the absence of pouch of Douglas obliteration in women with placenta previa (the cut-off value, AUC, PPV, and NPV were 10°, 0.89, 68.0%, and 94.3%, respectively) [5]. We examined the usefulness of the cervical canal angle to predict pouch of Douglas obliteration in 39 women with posterior placenta previa included in this study population, according to Matsuzaki et al.’s method. The cut-off value in our study population was 12°, as opposed to 10° in the above-mentioned study. This sensitivity, specificity, PPV, NPV, and AUC for this cut-off value were 50.0%, 100.0%, 100.0%, 91.6%, and 0.68, respectively. No significant difference was observed in the AUCs of the CIA and cervical canal angle (0.83 versus 0.68, p = 0.10) (Fig 2). However, the present study only included women with posterior placenta previa; hence, our results cannot be compared directly with those of the previous study. However, although there was no significant difference in the AUCs between the CIA and cervical canal angle, it is noteworthy that the performance of the CIA may be superior to that of the cervical canal angle, and the inter-rater reliability of the CIA was higher than that of the cervical canal angle. There are two possible explanations for this observation. First, the CIA is calculated relative to the anatomical conjugate line based on pelvimetry, which is influenced by maternal posture and surrounding soft tissues, such as the subcutaneous fat, to a considerably lesser degree than is the cervical canal angle. Second, MRI scans for placental evaluation do not always include the entire broad of the back, which is necessary to measure the cervical canal angle described by Matsuzaki et al. [5]. Accurate risk stratification of women with posterior placenta previa, whose posterior pelvic cavity is adherent to the surrounding organs, may facilitate clinical decision-making for hemostasis during cesarean section and transfer to higher-level medical facilities. If the CIA is higher than the cut-off value, women with posterior placenta previa may be considered for transfer to higher-level medical facilities, where bleeding can be controlled without procedures that require exteriorization of the uterus (e.g., uterine artery embolism) [24]. Moreover, identifying women with posterior placenta previa at a high risk of pouch of Douglas obliteration based on the CIA could also enable appropriate counseling with respect to increased surgical morbidity. Although performing the cesarean section was challenging, intraoperative complications did not occur in the six cases in which pouch of Douglas obliteration was observed. Preparation for surgery was adequate in all cases. However, an objective evaluation of pouch of Douglas obliteration using the CIA may have led to a reconfirmation of the preoperative difficulty of the surgery and a more straightforward explanation of the difficulty of the surgery to patients and their families.

This study has several limitations. First, extrapolating our results to the general population may be difficult because it is difficult to draw conclusions on the relationship between CIA and obliteration of the pouch of Douglas in this study due to the sample size and single center setting. The positive predictive value and sensitivity for predicting pouch of Douglas obliteration in this study were relatively low. As a result, it was challenging to accurately predict pouch of Douglas obliteration using CIA. However, the negative predictive value was very high at 92.9%, which may be useful in ruling out pouch of Douglas obliteration.

Therefore, a large-scale, multicenter, prospective cohort study is required to confirm these results in the general population. Second, MRI scans are generally not performed in cases of placenta previa, unless an adherent placenta is suspected; therefore, the results of this study should be interpreted with caution. Third, some potential risk factors that affected or biased the CIA, such as fetal weight, uterine myoma, and uterine adenomyosis, were not evaluated, which may have influenced the study results [5]. Fourth, the timing of the MRI depended on the patient’s condition. Therefore, the differences in MRI timings might induce differences in cervical angles and create new bias. Finally, patients with anterior placenta previa were not included in this study, since only seven women with anterior placenta previa underwent MRI for the preoperative assessment of PAS during the study period. Thus, we were unable to examine cases of anterior previa placenta due to the small sample size. The difference in the ease of extension of growth of the lower uterine segment between anterior and posterior placenta previa is well known [25, 26]; therefore, the cut-off value should be evaluated considering the location of the placenta. In the future, we endeavor to conduct another study on the same topic in patients with anterior placenta previa. We would also like to score the severities of pouch of Douglas obliteration and examine the relationship between that score and the CIA including both anterior and posterior placenta previa.

## Conclusions

CIA may have a high ability to rule out an obliteration of the pouch of Douglas. It may also be a useful indicator in the surgical management of women with posterior placenta previa. However, caution should be exercised when generalizing the results of this study because of the small sample size, which makes the results prone to bias.

## Supporting information

S1 Fig Ultrasound image showing a uterus in the retroflexed position. (TIF)

S2 Fig Ultrasound image showing a uterus in the anteverted position. (TIF)

S3 Fig Ultrasound image showing a uterus in the retroverted position. (TIF)

S1 File (XLSX)

## References

[1] Rosenberg T, Pariente G, Sergienko R, Wiznitzer A, Sheiner E. Critical analysis of risk factors and outcome of placenta previa. Arch Gynecol Obstet. 2011;284: 47–51. doi: 10.1007/s00404-010-1598-7 20652281

[2] Fan D, Xia Q, Liu L, Wu S, Tian G, Wang W, et al. The incidence of postpartum hemorrhage in pregnant women with placenta previa: a systematic review and meta-analysis. PLoS One. 2017;12: e0170194. doi: 10.1371/journal.pone.0170194 28107460 PMC5249070

[3] Benaglia L, Candotti G, Papaleo E, Pagliardini L, Leonardi M, Reschini M, et al. Pregnancy outcome in women with endometriosis achieving pregnancy with IVF. Hum Reprod. 2016;31: 2730–2736. doi: 10.1093/humrep/dew210 27664955

[4] Zullo F, Spagnolo E, Saccone G, Acunzo M, Xodo S, Ceccaroni M, et al. Endometriosis and obstetrics complications: a systematic review and meta-analysis. Fertil Steril. 2017;108: 667–672.e5. doi: 10.1016/j.fertnstert.2017.07.019 28874260

[5] Matsuzaki S, Okada A, Endo M, Nagase Y, Nakagawa S, Hiramatsu K, et al. Horizontal cervix as a novel sign for predicting adhesions on the posterior extrauterine wall in cases of placenta previa. J Clin Med. 2019;8: E2141. doi: 10.3390/jcm8122141 31817169 PMC6947443

[6] Hasegawa J, Matsuoka R, Ichizuka K, Mimura T, Sekizawa A, Farina A, et al. Predisposing factors for massive hemorrhage during Cesarean section in patients with placenta previa. Ultrasound Obstet Gynecol. 2009;34: 80–84. doi: 10.1002/uog.6426 19565529

[7] Jauniaux E, Bhide A. Prenatal ultrasound diagnosis and outcome of placenta previa accreta after cesarean delivery: a systematic review and meta-analysis. Am J Obstet Gynecol. 2017;217: 27–36. doi: 10.1016/j.ajog.2017.02.050 28268196

[8] Nagase Y, Matsuzaki S, Endo M, Hara T, Okada A, Mimura K, et al. Placenta previa with posterior extrauterine adhesion: clinical features and management practice. BMC Surg. 2021;21: 10. doi: 10.1186/s12893-020-01027-9 33407322 PMC7789541

[9] Arakaki T, Matsuoka R, Takita H, Oba T, Nakamura M, Sekizawa A. The routine use of prophylactic Bakri balloon tamponade contributes to blood loss control in major placenta previa. Int J Gynaecol Obstet. 2021;154: 508–514. doi: 10.1002/ijgo.13589 33421119

[10] Matsubara S, Kuwata T, Baba Y, Usui R, Suzuki H, Takahashi H, et al. A novel ’uterine sandwich’ for haemorrhage at caesarean section for placenta praevia. Aust N Z J Obstet Gynaecol. 2014;54: 283–286. doi: 10.1111/ajo.12184 24506478

[11] Arduini M, Epicoco G, Clerici G, Bottaccioli E, Arena S, Affronti G. B-Lynch suture, intrauterine balloon, and endouterine hemostatic suture for the management of postpartum hemorrhage due to placenta previa accreta. Int J Gynaecol Obstet. 2010;108: 191–193. doi: 10.1016/j.ijgo.2009.10.007 19945698

[12] Pala Ş, Atilgan R, Başpınar M, Kavak EC, Yavuzkir S, Akyol A, et al. Comparison of results of Bakri balloon tamponade and caesarean hysterectomy in management of placenta accreta and increta: a retrospective study. J Obstet Gynaecol. 2018;38(2): 194–199. doi: 10.1080/01443615.2017.1340440 28903630

[13] Macario S, Chassang M, Novellas S, Baudin G, Delotte J, Toullalan O, et al. The value of pelvic MRI in the diagnosis of posterior cul-de-sac obliteration in cases of deep pelvic endometriosis. AJR Am J Roentgenol. 2012;199: 1410–1415. doi: 10.2214/AJR.11.7898 23169738

[14] Ahn KH, Lee EH, Cho GJ, Hong SC, Oh MJ, Kim HJ. Anterior placenta previa in the mid-trimester of pregnancy as a risk factor for neonatal respiratory distress syndrome. PLoS One. 2018;13: e0207061. doi: 10.1371/journal.pone.0207061 30388184 PMC6214571

[15] Jansen C, de Mooij YM, Blomaard CM, Derks JB, van Leeuwen E, Limpens J, et al. Vaginal delivery in women with a low-lying placenta: a systematic review and meta-analysis. BJOG. 2019;126: 1118–1126. doi: 10.1111/1471-0528.15622 30663270

[16] Ohkuchi A, Hirashima C, Takahashi K, Suzuki H, Matsubara S. Prediction and prevention of hypertensive disorders of pregnancy. Hypertens Res. 2017;40: 5–14. doi: 10.1038/hr.2016.107 27534740

[17] Minakami H, Maeda T, Fujii T, Hamada H, Iitsuka Y, Itakura A, et al. Guidelines for obstetrical practice in Japan: Japan Society of Obstetrics and Gynecology (JSOG) and Japan Association of Obstetricians and Gynecologists (JAOG) 2014 edition. J Obstet Gynaecol Res. 2014;40: 1469–1499. doi: 10.1111/jog.12419 24888907

[18] Seracchioli R, Raimondo D, Del Forno S, Leonardi D, De Meis L, Martelli V, et al. Transvaginal and transperineal ultrasound follow-up after laparoscopic correction of uterine retrodisplacement in women with posterior deep infiltrating endometriosis. Aust N Z J Obstet Gynaecol. 2019;59: 288–293. doi: 10.1111/ajo.12882 30136296

[19] Di Donato N, Seracchioli R. How to evaluate adenomyosis in patients affected by endometriosis. Minim Invasive Surg. 2014;2014: 507230. doi: 10.1155/2014/507230 25197569 PMC4146361

[20] Perkins NJ, Schisterman EF. The inconsistency of "optimal" cutpoints obtained using two criteria based on the receiver operating characteristic curve. Am J Epidemiol. 2006;163: 670–675. doi: 10.1093/aje/kwj063 16410346 PMC1444894

[21] Tissot M, Lecointre L, Faller E, Afors K, Akladios C, Audebert A. Clinical presentation of endometriosis identified at interval laparoscopic tubal sterilization: prospective series of 465 cases. J Gynecol Obstet Hum Reprod. 2017;46: 647–650. doi: 10.1016/j.jogoh.2017.05.003 28526518

[22] Sanders RC, Parsons AK. Anteverted retroflexed uterus: a common consequence of cesarean delivery. AJR Am J Roentgenol. 2014;203: W117–W124. doi: 10.2214/AJR.12.10403 24951223

[23] Matsuzaki S, Nagase Y, Ueda Y, Lee M, Matsuzaki S, Maeda M, et al. The association of endometriosis with placenta previa and postpartum hemorrhage: a systematic review and meta-analysis. Am J Obstet Gynecol MFM. 2021;3: 100417. doi: 10.1016/j.ajogmf.2021.100417 34098177

[24] Yuan Q, Jin Y, Chen L, Ling L, Bai XM. Prophylactic uterine artery embolization during cesarean delivery for placenta previa complicated by placenta accreta. Int J Gynaecol Obstet. 2020;149: 43–47. doi: 10.1002/ijgo.13072 31778209

[25] Ancona S, Chatterjee M, Rhee I, Sicurenza B. The mid-trimester placenta previa: a prospective follow-up. Eur J Radiol. 1990;10: 215–216. doi: 10.1016/0720-048x(90)90142-x 2192886

[26] Anderson NG, Wells SW, Allan RB. Placental compressibility: implications for indirect diagnosis of posterior placenta previa. Obstet Gynecol. 1992;79: 398–402. doi: 10.1097/00006250-199203000-00015 1738523

