# Supplementary figures and images for: Utility of the angle between the cervical canal and the anatomical conjugate line for predicting pouch of Douglas obliteration in patients with posterior placenta previa
Source: PLoS One. 2023 Aug 17;18(8):e0290244. doi: 10.1371/journal.pone.0290244 (PMC10434862; doi:10.1371/journal.pone.0290244)

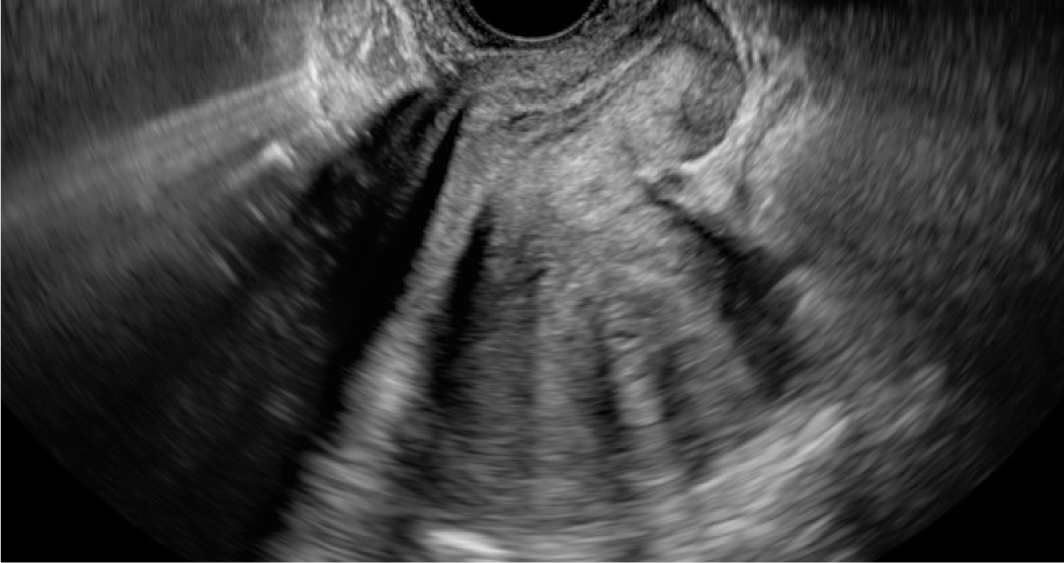

Supplement: S1 Fig — (TIF) [file pone.0290244.s001.tif]

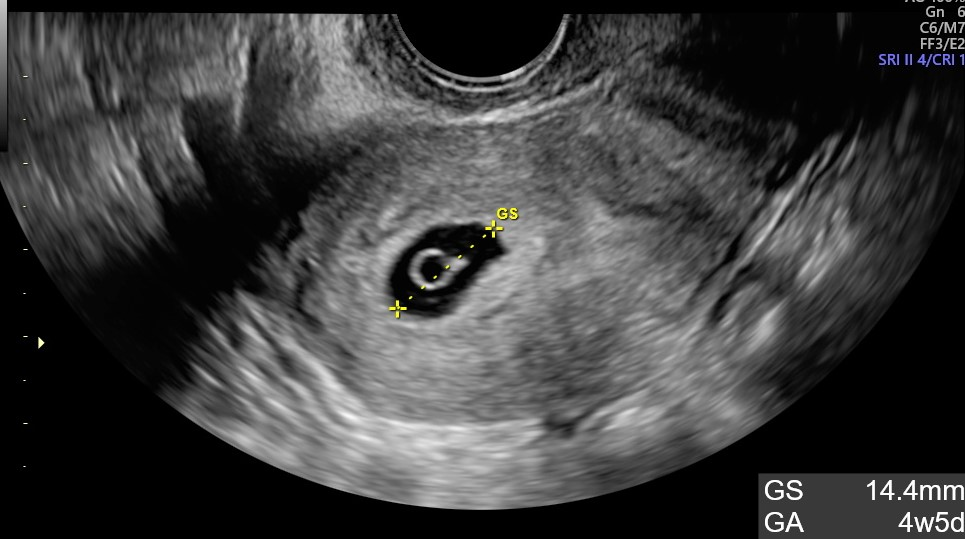

Supplement: S2 Fig — (TIF) [file pone.0290244.s002.tif]

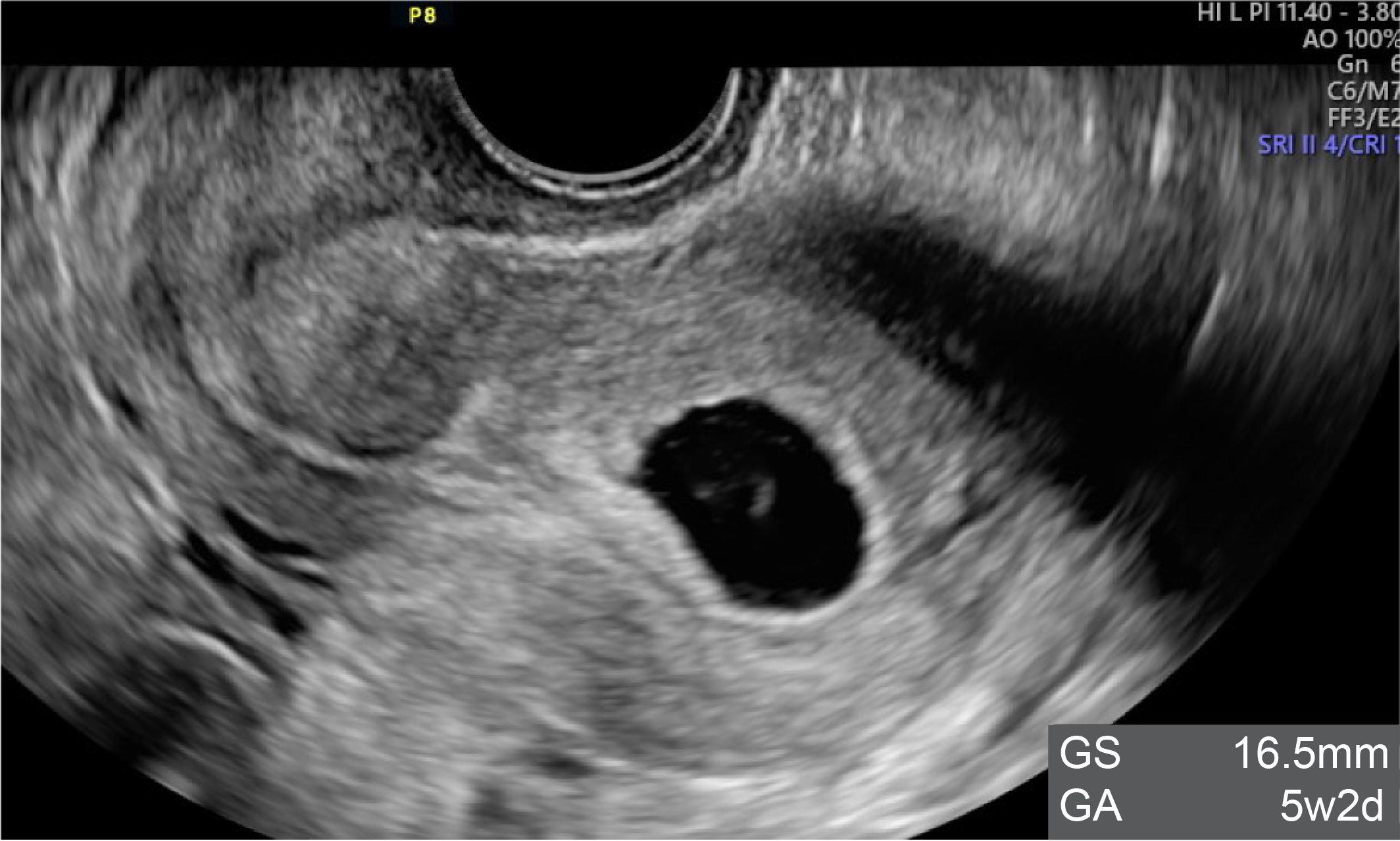

Supplement: S3 Fig — (TIF) [file pone.0290244.s003.tif]
